# Concordance of copy number loss and down-regulation of tumor suppressor genes: a pan-cancer study

**DOI:** 10.1186/s12864-016-2904-y

**Published:** 2016-08-22

**Authors:** Min Zhao, Zhongming Zhao

**Affiliations:** 1 School of Engineering, Faculty of Science, Health, Education and Engineering, University of the Sunshine Coast, Maroochydore DC, QLD 4558 Australia; 2 Department of Biomedical Informatics, Vanderbilt University School of Medicine, Nashville, TN 37203 USA; 3 Department of Cancer Biology, Vanderbilt University School of Medicine, Nashville, TN 37232 USA; 4 Department of Psychiatry, Vanderbilt University School of Medicine, Nashville, TN 37212 USA; 5 Center for Precision Health, School of Biomedical Informatics, The University of Texas Health Science Center at Houston, Houston, TX USA

**Keywords:** Tumor suppressor gene, Pan-cancer, Copy number variation, Copy number loss, Gene expression

## Abstract

**Background:**

Tumor suppressor genes (TSGs) encode the guardian molecules to control cell growth. The genomic alteration of TSGs may cause tumorigenesis and promote cancer progression. So far, investigators have mainly studied the functional effects of somatic single nucleotide variants in TSGs. Copy number variation (CNV) is another important form of genetic variation, and is often involved in cancer biology and drug treatment, but studies of CNV in TSGs are less represented in literature. In addition, there is a lack of a combinatory analysis of gene expression and CNV in this important gene set. Such a study may provide more insights into the relationship between gene dosage and tumorigenesis. To meet this demand, we performed a systematic analysis of CNVs and gene expression in TSGs to provide a systematic view of CNV and gene expression change in TSGs in pan-cancer.

**Results:**

We identified 1170 TSGs with copy number gain or loss in 5846 tumor samples. Among them, 207 TSGs tended to have copy number loss (CNL), from which fifteen CNL hotspot regions were identified. The functional enrichment analysis revealed that the 207 TSGs were enriched in cancer-related pathways such as P53 signaling pathway and the P53 interactome. We further performed integrative analyses of CNV with gene expression using the data from the matched tumor samples. We found 81 TSGs with concordant CNL events and decreased gene expression in the tumor samples we examined. Remarkably, seven TSGs displayed concordant CNL and gene down-regulation in at least 50 tumor samples: *MTAP* (212 samples), *PTEN* (139), *MCPH1* (85), *FBXO25* (67), *SMAD4* (64), *TRIM35* (57), and *RB*1 (54). Specifically to *MTAP*, this concordance was found in 14 cancer types, an observation that is not much reported in literature yet. Further network-based analysis revealed that these TSGs with concordant CNL and gene down-regulation were highly connected.

**Conclusions:**

This study provides a draft landscape of CNV in pan-cancer. Our findings of systematic concordance between CNL and down-regulation of gene expression may help better understand the TSG biology in tumorigenesis and cancer progression.

**Electronic supplementary material:**

The online version of this article (doi:10.1186/s12864-016-2904-y) contains supplementary material, which is available to authorized users.

## Background

Cancer is characterized by unconstrained cell proliferation. In the normal cell, there is precise control of cell division such as cell cycle check points [1]. In cellular system, tumor suppressor genes (TSGs) are important guardian genes that protect a normal cell from one step on the path to uncontrolled growth [2, 3]. In cancer cells, TSGs may lose their normal functions because of mutations occur at its critical sites. For single nucleotide or small insertions/deletions (indels), these mutations often lead to truncation of transcripts or proteins, including nonsense mutations, splicing site mutations, or frameshift mutations. Similar effects can be caused by larger scale mutations, such as copy number variations (CNVs), gene fusions, or structural variants (SVs) [4, 5]. The mutated TSGs often coordinate with oncogenes for cancer progression [4, 6, 7]. Therefore, the identification and understanding of TSGs have profound influence to develop the diagnosis biomarkers and effective drugs for cancer therapies.

CNVs are the variable number of DNA fragments in the human genome. Their lengths typically range from a kilo base pairs to a mega base pairs [8]. CNVs are divided into two major groups: copy number loss (CNL) and copy number gain (CNG). CNL denotes the decreased gene (or sequence fragment) copies in the genome while CNG denotes the gain of additional gene copies in the human genome. With the development of high throughput technologies such as Comparative genomic hybridization (CGH) array and next-generation sequencing, a very large number of CNVs, as well as other types of mutations and genomics data (e.g., gene expression) have been unveiled, especially in cancer genomes [9, 10]. This allows us to systematically study cancer mutation signatures, heterogeneity, and other molecular features [11]. For CNV, such deleted or duplicated DNA fragments often have profound effects on gene expression, which subsequently affects gene’s function [12].

Despite a number of studies have explored CNVs and gene expression in various cancers [13], there has been no systematic study of the features in TSGs yet. Moreover, the results from single cancer type may not be representative in other types of cancer, or they may vary among the subtypes of the cancer. To overcome these limitations, we conducted a pan-cancer CNV analysis on TSGs to explore the landscape of CNV features and cross-validate some observations. This study may help us better elucidate the relationship between CNV and gene expression change in this important gene category in cancer.

## Methods

### The curated TSGs from thousands of literatures

To conduct a systematic CNV survey of TSGs, we downloaded all the 1207 curated human TSGs from TSGene database in a plain text format with all the Entrez Gene IDs and official symbols (version 2.0) [2]. In this version of TSGene database, there were 1088 protein-coding and 198 non-coding TSGs. All these TSGs were manually curated from over 9000 PubMed abstracts by us. To annotate TSGs with CNVs, it requires the genomic location for mapping. Therefore, we downloaded the corresponding RefSeq mapping information for TSGs from RefSeq database. We implemented an in-house script to extract all the genomic location information from the completed human genome RefSeq sequences (accession number starting with NC). In total, 1207 TSGs were annotated with accurate genomic locations in GRCH 38.

### The pan-cancer CNV data from The Cancer Genome Atlas (TCGA)

To explore the CNVs in pan-cancer systematically, we downloaded all the prepared TCGA CNV data with the GRCH 38 genomic coordinates from the Catalogue of Somatic Mutations in Cancer (COSMIC) database (V73). When integrating TCGA data, COSMIC introduced a few thresholds to define the copy number loss and gain. CNG was obtained by the following criteria: (the average genome ploidy < =2.7 AND total DNA segment copy number > =5) OR (average genome ploidy >2.7 AND total DNA segment copy number > =9). Similarly, the criteria for CNL were: (the average genome ploidy < =2.7 AND total DNA segment copy number =0) OR (average genome ploidy >2.7 AND total DNA segment copy number < (average genome ploidy – 2.7)). In this study, we followed COSMIC criteria and overlapped all the CNV regions with TSGs using the GRCH 38 coordinates. By intersecting all the CNV gain and loss information to all the 1207 TSGs with GRCH 38 coordinates, we annotated 1170 TSGs with precise gain or loss information. For each cancer type, we calculate the number of samples with CNL and CNG, respectively. Since TSGs are often in loss-of-function in cancer progression, we pulled out those TSGs with higher frequency of CNLs than that with CNGs. Specifically, we set a cut-off of 2 to filter out those TSGs without having at least twice of tumor samples with CNLs as tumor samples with CNGs. This process resulted in 207 TSGs with the evidence of an overall loss of CNVs. These genes were used for the following gene expression analysis.

### Gene expression analysis for TSGs with CNL

To check the CNV-correlated gene expression changes on TSGs, we downloaded the TCGA pan-cancer gene expression data from the COSMIC database (V73). In this study, we focused on only those gene expression changes in the matched TCGA samples with TSG CNLs. For the gene expression quantification, COSMIC started from FPKM calculated using trimmed short reads generate by the RNA-Seq platform and the RSEM quantification results from the RNAseq V2 platform. Here, FPKM denotes Fragments Per Kilobase of transcript per Million mapped reads, which is used to indicate the relative expression of a transcript. And RSEM is one of the popular measures for accurate transcript quantification of RNA-Seq data. The average and the standard deviation of expression were computed using those tumor samples that are diploid for each corresponding gene.

The standard Z score was used to characterize whether a TSG is over or under expressed. The Z-score with absolute value 2 was used as the threshold value. The Z-score over 2 was defined as over expression while the Z-score less than −2 represented the decreased gene expression. For those 81 TSGs with CNL-associated gene expression change, we further systematically examined their somatic CNV patterns in pan-cancer of TCGA samples using cBio portal [14].

### Sub-network extraction for the TSGs with high frequency CNLs

To explore the relevant biological mechanisms related to TSGs with frequently observed CNLs and consistent gene down-regulation, we extracted a PPI network to connect 81 TSGs with the remaining human genes. To this goal, we started from a non-redundant human interactome extracted from the Pathway Commons database [15, 16], containing 3629 proteins and 36,034 PPIs. It is worth noting that this integrated human interactome is based on well-curated pathway databases (HumanCyc, Reactome, and KEGG pathway database [17]). Therefore, those links in the interactome have biological meaning rather than physical interactions. Based on the pathway-based interactions, we used the similar approach implemented in our previous study to extract a sub-network related to our 81 TSGs [16, 18, 19]. In this sub-network extraction strategy, all the 81 seed TSGs were overlapped to the human pathway-based interactome. Then, a sub-network with the maximum number of the seed TSGs was formed by connecting each TSG through the shortest path. To characterize the function of the network, we relied on the network topological properties (degree and shortest path) calculated from the network. In practice, we utilized NetworkAnalyzer plugin in Cytoscape 2.8 to compute topological properties in the TSG network [20]. The degree is defined as the number of direct connections of each node with other nodes in the TSG network [21, 22]. The network layout was conducted based on Cytoscape 2.8 [20].

## Results

### Genomic regions with frequent copy number loss in TSGs in multiple cancer types

**Fig. 1:**
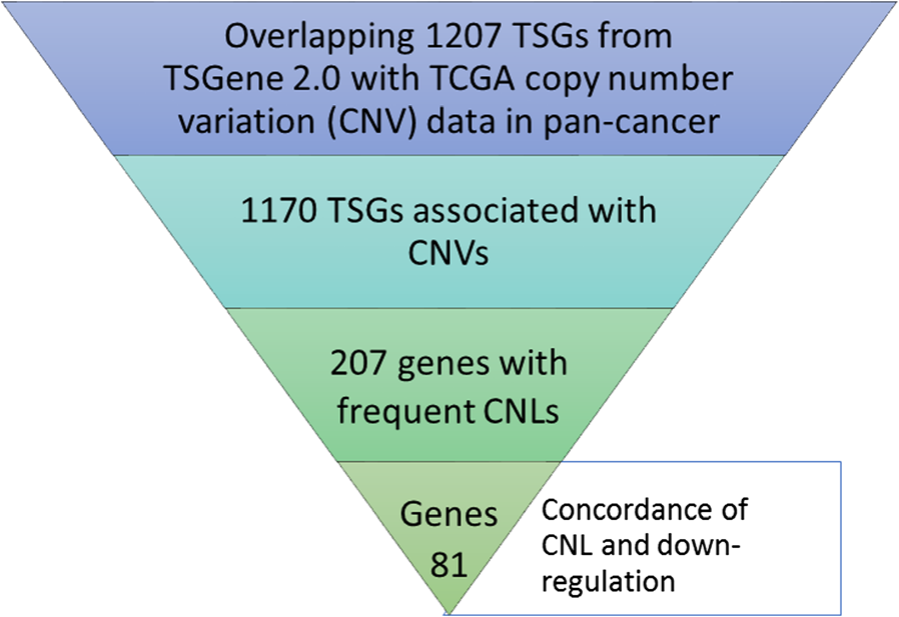
Pipeline for the identification of concordant copy number loss and down-regulation of tumor suppressor genes in human cancer. This figure shows the pipeline for identifying the tumor suppressor genes (TSGs) with concordant copy number variations (CNVs) and gene expression. It involves four main steps. 1) Downloading TSGs from the TSGene 2.0 database and overlapping to the TCGA pan-cancer CNV data. 2) The resulted 1170 TSGs with CNV overlapping information were further extracted and calculated the precise copy number gain (CNG) and loss (CNL). 3) Based on the number of samples with CNGs and CNLs in the pan-cancer CNV data, we collected 207 TSGs with frequent CNLs. 4) Using the gene expression data from the matched TCGA cancer samples, we identified 81 TSGs with consistent CNLs and decreased gene expression in the same samples

To systematically survey the somatic CNVs in TSGs, our pipeline started with a list of 1207 human TSGs from the TSGene 2.0 database [2, 23] (Fig. 1). These genes have multiple lines of evidence in literature and other data, and have been manually curated. To provide an unbiased global view of CNVs in major types of cancer, we overlapped all these 1207 TSGs with the somatic CNVs identified from TCGA, which is the largest cancer genomics data source. This resulted in a list of non-redundant 1170 TSGs, which are annotated with CNVs (Additional file 1: Table S1). However, the majority CNVs are not informative due to the lack of matched control tissue. In this study, we only focused on those TSGs with precise gain or loss data using the normal tissue as control (see Methods). By counting the number of samples with gain or loss of gene copies, we set a threshold to prioritize most informative CNV events for TSGs. Since TSGs typically play their roles by loss-of-function, we used only those TSGs that tend to have copy number loss. To this end, we required CNVs were observed in at least as twice the samples with copy number loss as those with copy number gain. The process resulted in a total of 207 TSGs. We named them as TSGs with CNL in cancer, and used them for the follow up functional enrichment and integrative analyses. The list is provided in Additional file 2: Table S2.

**Fig. 2:**
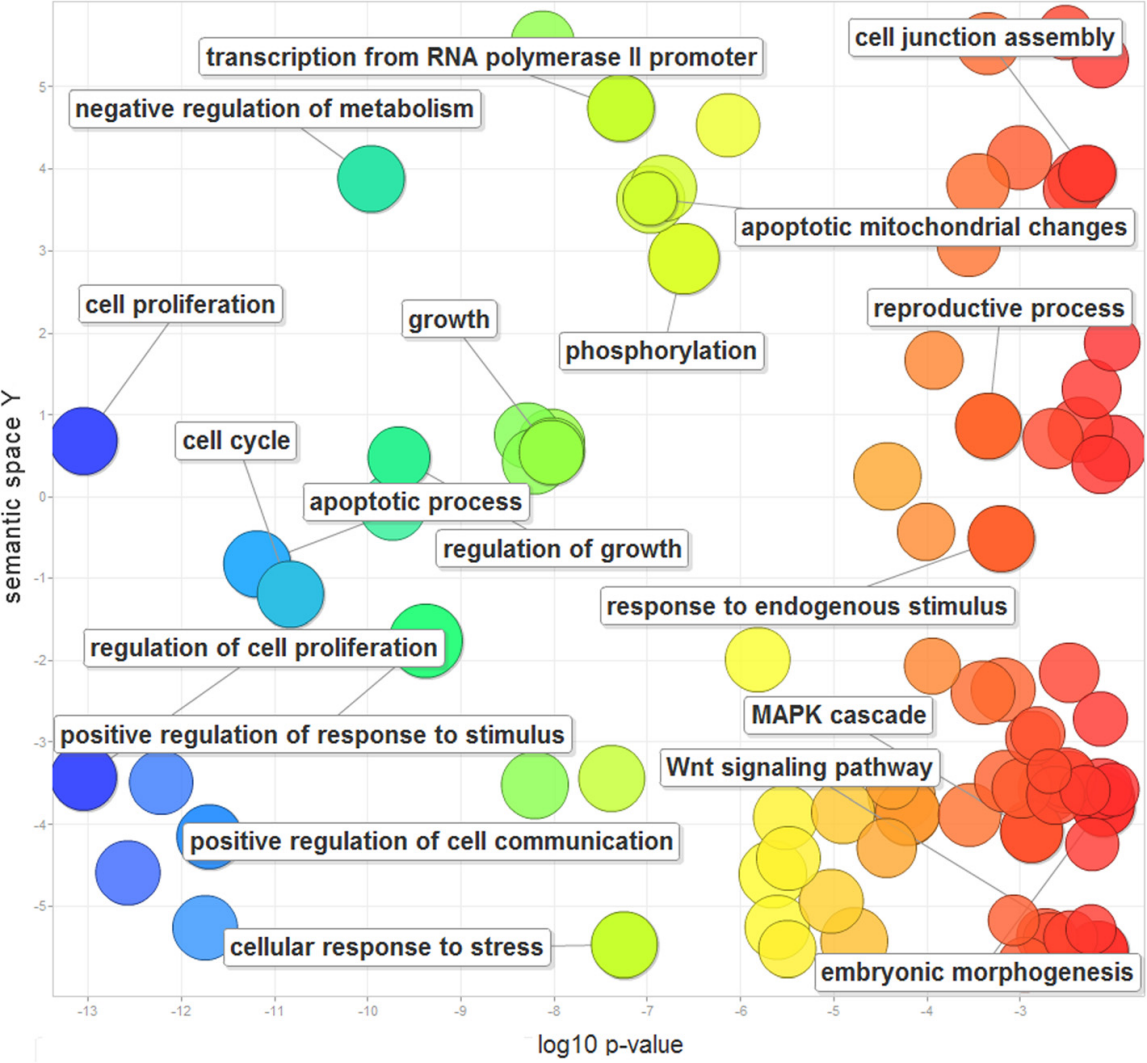
Gene Ontology (GO) analysis of 207 human tumor suppressor genes (TSGs) with frequent copy number losses (CNLs). The scatterplot shows the GO clusters for the 207 TSGs with CNLs in a two-dimensional space derived by applying multidimensional scaling to a matrix of the GO terms' semantic similarities. Bubble color represents the frequency of the GO term in the GOA database (more general terms are toward red). Bubble size indicates the log of corrected p-value (the smaller corrected p-value, the larger bubble)

We performed functional enrichment analysis of these 207 genes using Gene Ontology (GO) terms as functional units. Figure 2 displays the main features of GO-related functional features, and their clusters. Overall, they are enriched with cell proliferation, apoptosis, cell cycle, and growth control; all are important features of cancer cells. The TSGs with CNL also have fundamental roles in development such as embryonic morphogenesis and reproduction. In addition, they involve in negative regulation of cell metabolism and protein phosphorylation. Some TSGs may influence cell communication, cell junction assembly, and response to the extracellular stimulus.

Interestingly, the 207 TSGs could highly cluster into fifteen chromosome regions. All these regions had the corrected enrichment *p*-values less than 0.01 (Table 1). Among the 15 regions, eight could be further clustered into three genomics locations: 3p21, 8p21-22, and 17p13.1-3. In 3p21, we found four enriched cytobands with a total of 27 TSGs. For example, the 3p21.3 cytoband is enriched with 14 TSGs (*CTDSPL, CYB561D2, LIMD1, MST1R, NPRL2, PTPN23, RASSF1, RBM5, RBM6, RHOA, SEMA3B, SEMA3F, TUSC2*, and *ZMYND10*). Another six TSGs (*CDCP1, LTF, MIR1226, SETD2, SMARCC1*, and *TDGF1*) were clustered in 3p21.31. The remaining 7 TSGs were located close to 3p21. These 27 TSGs in 3p21 had CNLs in 129 TCGA samples covering 20 different cancer types. Specific to the tissue site, there were 27, 20, 15, 15, and 11 samples from lung, central nervous systems, kidney, breast, and large intestine, respectively. A similar observation of the high frequency of loss involving the short arm of chromosome 3 was reported as a tumor suppressor locus in a variety of histologically different neoplasms more than twenty years ago [24, 25]. However, our survey provides precise locations in various cancer samples.

**Table 1:** The 15 genomics regions associated with 207 tumor suppressor genes (TSGs) with frequent copy number losses (CNLs)

Cytoband	*p*-value	q-value	# TSGs	TSG list
3p21.3	5.98E-19	3.48E-16	14	*CTDSPL, CYB561D2, LIMD1, MST1R, NPRL2, PTPN23, RASSF1, RBM5, RBM6, RHOA, SEMA3B, SEMA3F, TUSC2, ZMYND10*
8p22	4.48E-09	9.78E-07	7	*CCAR2, DLC1, LZTS1, MIR383, MTUS1, SOX7, ZDHHC2*
11p15.5	5.05E-09	9.78E-07	10	*CDKN1C, H19, MIR210, MIR483, NUP98, RNH1, SIRT3, TRIM3, TSPAN32, TSSC4*
17p13.1	5.30E-08	7.71E-06	9	*ALOX15B, BCL6B, GABARAP, MIR195, MIR497, TNK1, TP53, XAF1, ZBTB4*
19p13.3	2.52E-06	2.93E-04	10	*AMH, DAPK3, DIRAS1, FZR1, GADD45B, PLK5, SIRT6, STK11, TCF3, TNFSF9*
8p21.3	3.45E-06	3.34E-04	5	*DOK2, MIR320A, PIWIL2, PPP3CC, RHOBTB2*
3p21.31	3.63E-05	3.01E-03	6	*CDCP1, LTF, MIR1226, SETD2, SMARCC1, TDGF1*
3p21.1	1.49E-04	1.02E-02	4	*ACY1, CACNA2D3, MIR135A1, MIRLET7G*
6q26	1.58E-04	1.02E-02	3	*MAP3K4, IGF2R, PACRG*
10q24-q25	2.09E-04	1.16E-02	2	*CHUK, MXI1*
8p21	2.19E-04	1.16E-02	3	*BNIP3L, EXTL3, TNFRSF10A*
17p13.3	2.40E-04	1.16E-02	5	*ALOX15, MNT, MYBBP1A, PAFAH1B1, VPS53*
22q13.31	2.62E-04	1.17E-02	4	*FBLN1, MIRLET7B, MIRLET7A3, PPARA*
9p21	7.46E-04	3.10E-02	3	*CDKN2A, CDKN2B, MTAP*
3p21	1.08E-03	4.19E-02	3	*GNAT1, MST1, PBRM1*

q-values were calculated by Benjamini-Hochberg multiple testing correction of the raw *p*-values, which were calculated by the hypergeometric test

On chromosome 8, the 8p22 locus contained 7 neighbouring TSGs (*CCAR2, DLC1, LZTS1, MIR383, MTUS1, SOX7*, and *ZDHHC2*), while another 8 TSGs (*BNIP3L, DOK2, EXTL3, MIR320A, PIWIL2, PPP3CC, RHOBTB2*, and *TNFRSF10A*) clustered at 8p21. These 15 TSGs at 8p21-22 had CNL detected in 219 TCGA patients. The cancer tissues that had most frequent CNLs in TSGs at this locus are breast (61 samples), lung (42 samples), large intestine (30 samples), ovary (23 samples), and prostate (11 samples). Another CNL hot region is at 17p13.1-3, which covers 14 TSGs, including the most studied TSG *TP53*. This region on chromosome 17 had detectable CNLs in a total of 50 TCGA tumor samples. Interestingly, the above three genomic regions with frequent CNLs in TSGs harbour not only well-known protein-coding TSGs such as *TP53*, but also six microRNAs (MIRLET7G, MIR135A1, MIR195, MIR320A, MIR383, and MIR497). By overlapping to TSGene database, we found all the six microRNAs are tumor suppressor microRNAs. Collectively, our systematic examination on CNL in TSG cluster regions provides precise information of such CNL in multiple cancers. The results may be useful for further studying the similar or different roles of CNL in differential cancer types as well as cancer heterogeneity.

### Correlation of CNL with gene expression decrease using the matched tumor samples

Through incorporating the gene expression change of the TCGA samples with the CNL on TSGs, we examined the correlation between CNL and TSG gene down-regulation. We utilized the Z-score to assess whether a TSG is up-regulated or down-regulated in specific TCGA samples. Here, Z-score is a transformation of the *p* value calculated by the formula as below:

$$ Z=\frac{x\mathit{\hbox{-}}\mu }{\sigma } $$

Where μ represents the mean expression of a gene across multiple TCGA samples; σ represents the standard deviation of the expression scores of the gene in TCGA samples. Specifically, we used the Z-score threshold value −2 to identify down-regulated TSGs in specific TCGA samples.

**Fig. 3:**
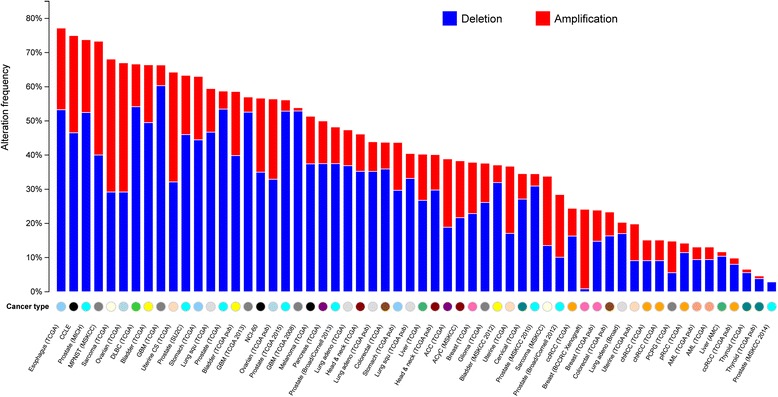
A pan-cancer global view of copy number variation (CNV) features based on 81 tumor suppressor genes (TSGs) with decreased gene expression potentially induced by copy number losses (CNLs)

After examining the same TCGA tumor samples for both expression and CNV loss, we found 81 TSGs that had concordant decreased gene expression and loss of gene copy numbers in tumor samples (Additional file 3: Table S3). The functional enrichment analyses revealed that the 81 TSGs are mainly associated with cancer-related pathways such as cell cycle (adjusted *P*-value = 1.15E-6) (Additional file 4: Table S4). Interestingly, they are also related to a number of cancer-related phenotypes such as hamartomatous polyposis (adjusted *P*-value = 1.05E-6) and intussusception (adjusted *P*-value = 3.98E-6). The CNV mutational patterns across multiple cancers are plotted in Fig. 2. In terms of their CNVs, these 81 TSGs are highly mutated. For example, in TCGA esophageal carcinoma cohort, there were 142 cases (77.2 %) that had at least one gene with copy number change (Additional file 5: Table S5). More than 50 % of the esophageal carcinoma patients had at least one deletion event in one of the 81 TSGs. The similar prevalence of copy number alteration (>60 % cases, including both CNLs and CNGs) was found in other 11 cancer datasets from 9 cancer types: metastatic prostate cancer, malignant peripheral nerve sheath tumor, sarcoma, ovarian serous cystadenocarcinoma, lymphoid neoplasm diffuse large B-cell lymphoma, bladder urothelial carcinoma, glioblastoma multiforme, uterine carcinosarcoma, and stomach adenocarcinoma. However, specific to CNLs, only the glioblastoma multiforme (GBM) cohort had over 60 % patients with CNLs (Fig. 3, the blue bar represent the CNLs in different cancer types). Although the other cancer cohorts also possess CNLs in the majority of the affected patients, a small portion of patients had CNGs rather than CNLs. This may imply the importance of the 81 TSGs in cancer progression of GBM via the massive copy number losses.

**Fig. 4:**
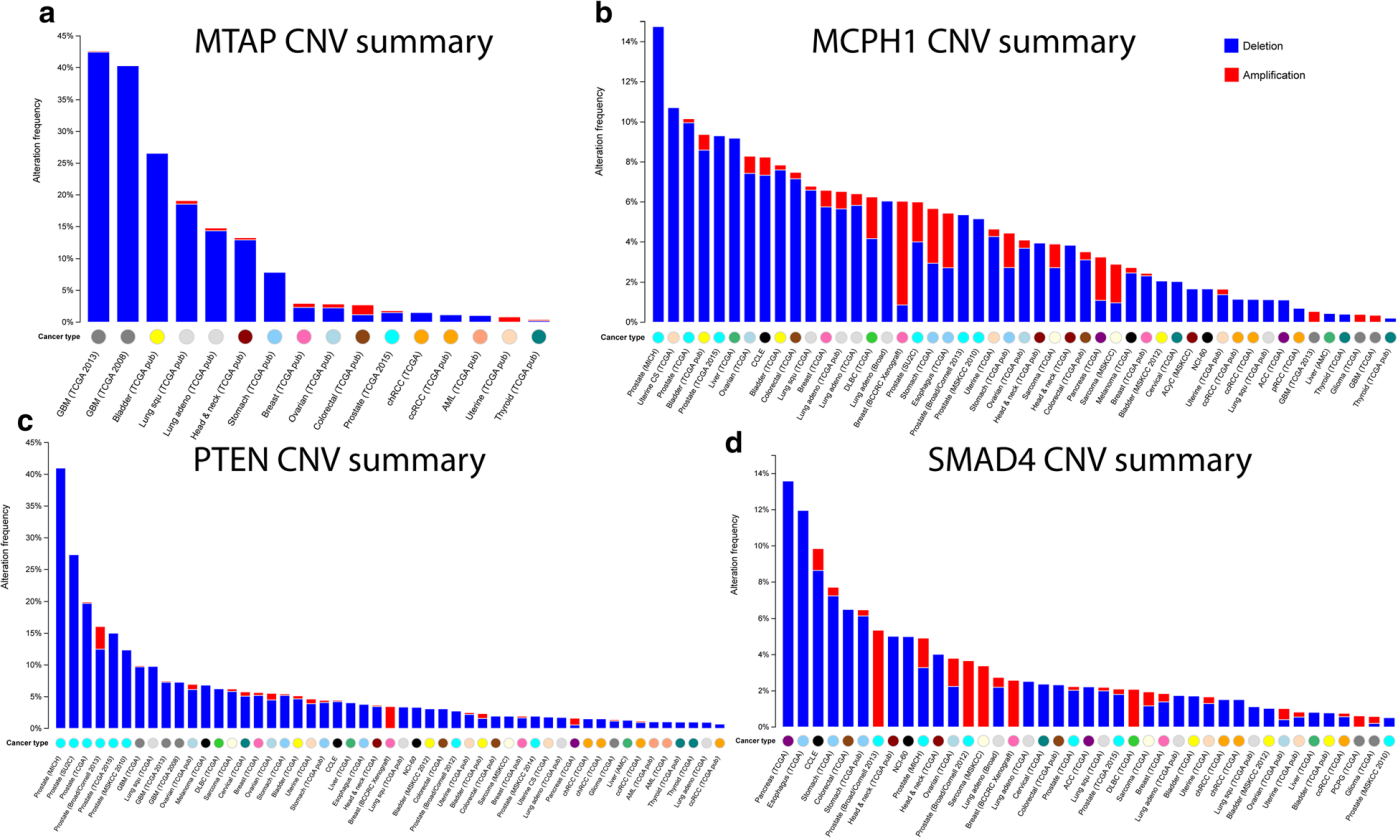
A pan-cancer view of copy number of variation (CNV) distribution in four tumor suppressor genes: *MTAP*, *MCPH1*, *PTEN*, and *SMAD4*. The CNV mutational landscape for (**a**) *MTAP*, (**b**) *MCPH1*, (**c**) *PTEN*, and (**d**) *SMAD4*

**Fig. 5:**
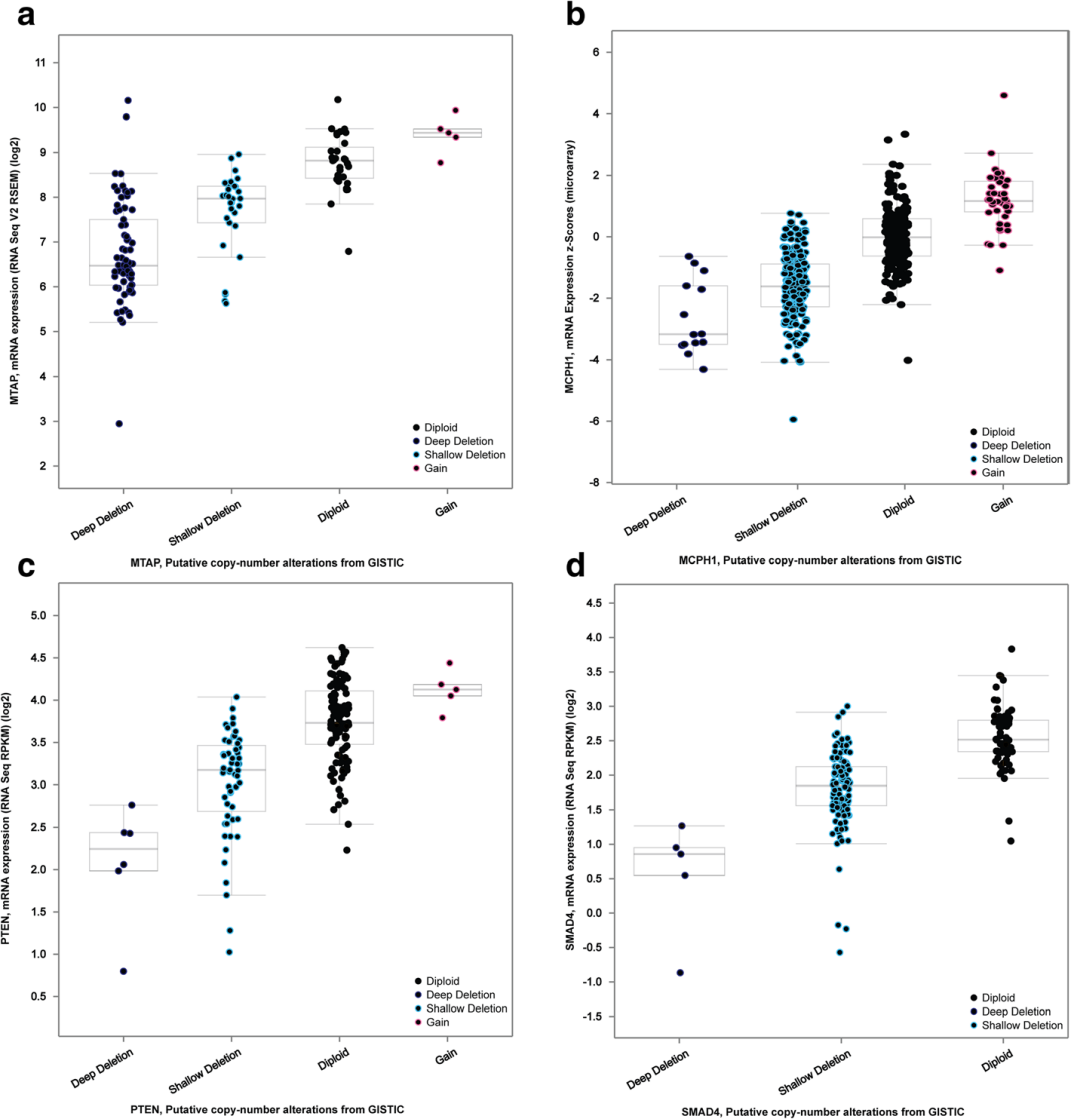
The correlation of copy number variation (CNV) and gene expression in four tumor suppressor genes: *MCPH1*, *MTAP*, *PTEN*, and *SMAD4*. **a** *MTAP* using TCGA glioblastoma data, (**b**) *MCPH1* using TCGA breast cancer data, (**c**) *PTEN* using TCGA lung squamous carcinoma (LUSC), and (**d**) *SMAD4* using TCGA colorectal cancer data

To further explore the potential CNL-induced gene expression change, we specifically checked four TSGs with the most frequently observed CNLs; all these genes were observed in more than 50 tumor samples. As shown in Fig. 4, TSG *MTAP* had CNL in more than 40 % cases in TCGA GBM cohort. The TSG *MCPH1* was deleted in more than 14 % patients in a prostate cancer dataset. *PTEN* showed similar frequent CNV loss in prostate cancer samples. The gene loss of *SMAD4* was prominent in the pancreatic cancer. Furthermore, we found consistent, low gene expression of these four genes in the tumor samples with CNL (Fig. 5). The results suggested that CNL might induce gene expression decrease as a common mechanism in cancer.

### A connected biological map of TSGs with concordant CNL and decreased gene expression

**Fig. 6:**
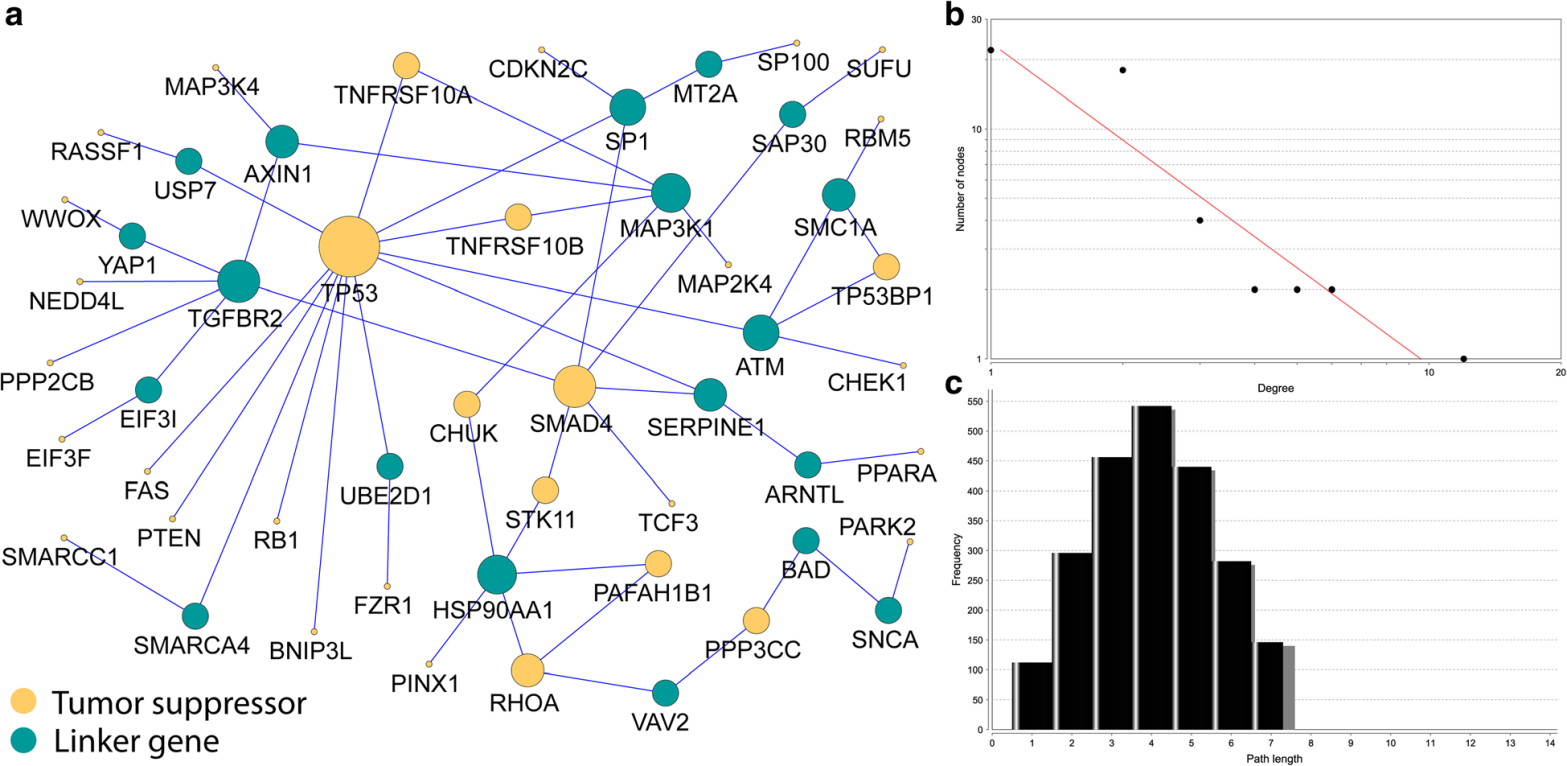
Reconstructed interaction map for the 81 tumor suppressor genes (TSGs) with decreased gene expression potentially induced by copy number losses (CNLs). **a** The network includes 35 genes (in yellow) from the 81 TSGs with decreased expression potentially induced by CNLs and 19 linker genes (in blue) that connect these 35 TSGs. The node size reflects the number of connection. A bigger size means more connections associated with the gene. **b** The degree distribution of the nodes (genes) in the network (**a**). **c** The distribution of the shortest path length

To further investigate the common functional regulation and enhance our understanding of the cellular events of these 81 TSGs with decreased expression and CNL, we conducted a pathway-based protein-protein interaction (PPI) analysis using the pathway annotation data from Pathway Commons database [15]. These reliable interactions are based on evidence from known biological pathways such as the KEGG and Reactome pathway databases. Therefore, this pathway-based interactome is useful for the pathway reconstruction because such pathways may avoid high-level noises, sparseness, and highly skewed degree distribution, which are often observed in physical interaction-based PPI networks. By applying the Klein-Ravi algorithm for module searching [18], we first mapped the 81 TSGs to the human pathway interactome. Then, a subnetwork was extracted, allowing to connect as many as the 81 TSGs as possible. The reconstructed TSG network contains 54 nodes and 56 links (Fig. 6a). Among the 54 nodes, 35 are from the 81 TSGs. The remaining 19 are the linker genes to bridge those TSGs so that a fully connected map could be built. The degrees of the nodes in this reconstructed map potentially follow a power law distribution *P(k) ~ k*^*-b*^, where *P(k)* is the probability that a node has connections with other *k* nodes and *b* is an exponent with an estimated value of 1.4 (Fig. 6b). Moreover, most of the genes in the network can be connected by three to five steps on average, as measure by the shortest path (Fig. 6c). These two topological features (degree and shortest path) indicated that most TSGs in this map were closely connected with high modularity. Considering the tight connection of the map, the nodes with multiple connections are likely to play critical and concordant roles to mediate biological regulation such as signalling transduction in cellular system. In the network, there are 7 nodes with four or more connections: *TP53* (12 connections), *SMAD4* (6), *TGFBR2* (6), *MAP3K1* (5), *HSP90AA1* (5), *ATM* (4), and *SP1* (4). It is interestingly that *TP53* is the node with most connection in the network. *SMAD4* is also in the centre of the map with six connections. In summary, our reconstructed map for the 81 TSGs with potential CNL-driven gene down-regulation contains some interesting features such as the TSGs with potential CNL-induced dysregulation.

## Discussion

This study revealed some important somatic mutational features of TSGs in multiple cancer types, particularly with respect to the CNVs and their effects on gene expression. Since the loss-of-function is the typical mechanism that TSGs involve in cancer initiation and progression, a large-scale change of gene copy number may induce gene expression alteration. In this scenario, a critical regulation change is that CNL in a TSG leads to the over-expression of its guardian genes. Although previous studies have explored the balance of germline CNVs and gene expression, there still lack of direct links of somatic CNVs on gene expression dosage compensation. In this study, we only focused on the concordant patterns between CNL and gene down-regulation because TSGs often play functions in a manner of loss-of-function. Our results only provided the insight of correlation between gene dosage and somatic CNV; more systematic examination of the expression quantitative trait locus may provide more depth on the relationship between CNV and gene expression.

This study was mainly based on the TCGA genomic data. The cohort size of some cancer types is relatively small (e.g., ~100 samples). A small sample size may filter out many low-frequency CNVs. In addition, TCGA mainly relies on the CGH array between normal and tumor tissues to characterize CNVs, which may lose signals outside of pre-designed probes. These undetected CNVs may also contribute to TSGs functionality on cancer progression. Another limitation in this study is that we only incorporate the protein-coding gene expression, not including non-coding gene expression. The further integration of large-scale CNV data and gene expression of noncoding RNA (microRNA and long non-coding RNA) may provide new insight into the roles of the non-coding TSGs.

In this study, we made an effort to construct a biological map for the genes with consistent CNL and gene down-regulation in cancer. Although the majority of genes in the reconstructed map are linked with each other, the size of the network is relatively small. Therefore, it has limited power to explore the overall network functions based on the topological features. For example, we found the degree of the network might follow the power-law distribution. This feature is different from the whole human PPI network, in which the majority nodes (genes) are sparsely connected with exponent *b* as 2.9 [26]. It is not sufficient to impose the scale-free properties on this constructed small network due to the small size. For the same reason, it is not good for us to define the hub nodes based on the high connectivity. Nevertheless, the nodes with multiple connections in our network should provide some clues for the common CNL events related to gene down-regulation. The further experimental validation may provide more insight into the potential molecular mechanisms for those CNL events that were detected in multiple cancers.

## Conclusions

In conclusion, our systematic exploration of copy number variations on human TSGs revealed that the copy number loss of TSGs cluster in a few genomics regions. These TSGs with frequent copy number loss often have profound roles in cancer-related pathways. The loss of copy number in a number of TSGs may contribute to the gene expression change involving tumorigenesis.

## Abbreviations

CNG, copy number gain; CNL, copy number loss; CNV, copy number variation; TCGA, The Cancer Genome Atlas; TSG, tumor suppressor gene

## Additional files

Additional file 1: Table S1. The 207 tumor suppress genes (TSGs) with frequent copy number losses (CNLs). (XLSX 18 kb) Additional file 2: Table S2. The enriched pathways and interactors for the 207 tumor suppress genes (TSGs) with frequent copy number loss (CNL). (XLSX 11 kb) Additional file 3: Table S3. The 81 tumor suppress genes (TSGs) with decreased gene expression potentially induced by copy number losses (CNLs). (XLSX 15 kb) Additional file 4: Table S4. Functional enrichment results of 81 tumor suppress genes (TSGs) with decreased gene expression potentially induced by copy number losses (CNLs). (XLSX 24 kb) Additional file 5: Table S5. The CNV frequency of 81 tumor suppress genes (TSGs) with decreased gene expression potentially induced by copy number losses (CNLs) in multiple cancers. (XLSX 12 kb)
